# Conservation and Recombination in the Genome Sequence of *Haemophilus influenzae* Type f WAPHL1

**DOI:** 10.1128/genomeA.00929-17

**Published:** 2017-09-21

**Authors:** Allen C. Bateman, Ailyn C. Perez-Osorio, Zhen Li, Michael Tran, Alexander L. Greninger

**Affiliations:** a Department of Laboratory Medicine, University of Washington, Seattle, Washington, USA; b Washington State Public Health Laboratory, Seattle, Washington, USA

## Abstract

We report here the second draft genome sequence of a bloodstream isolate of *Haemophilus influenzae* serotype f. Three discrete 3.1- to 7.8-kb sites contained 80% of the variability in the genome, consistent with recombination in known virulence factors.

## GENOME ANNOUNCEMENT

The incidence of invasive *Haemophilus influenzae* type b infection has decreased dramatically following vaccine introduction, but nontypeable and type f *H. influenzae* strains have increased in the United States and Sweden (1, 2). We report here a draft genome sequence of a bloodstream isolate of *H. influenzae* type f. To our knowledge, this is the second full genome of *H. influenzae* type f, which allows for a comparison to the previously published genome (3).

A man in his 50s presented with hypothermia, acidemia, and shock and was found to have hypoxemic respiratory failure. Aerobic and anaerobic bottles grew *H. influenzae* and a coagulase-negative *Staphylococcus* sp. The *H. influenzae* isolate tested beta-lactamase negative, and a workup at the Washington State Public Health laboratories showed that the *H. influenzae* isolate was type f, biotype I.

The DNeasy blood and tissue kit (Qiagen, Valencia, CA) was used to extract *H. influenzae* isolate DNA. The shotgun DNA library was prepared using a Nextera XT kit and sequenced via an Illumina MiSeq sequencer (4). A total of 2,379,348 2 × 250-bp paired-end reads were adapter and quality trimmed (Q30) using cutadapt, repaired using pairfq, *de novo* assembled using SPAdes, and annotated via Prokka (5–7). The genome assembly yielded 35 contigs measuring a total of 1,806,861 bp, with an *N*_50_ value of 137,750 bp and a GC content of 37.9%. A total of 1,709 coding sequences were annotated, and no plasmids were detected. The closest sequence in the NCBI NT database according to a BLASTN search and average nucleotide identity according to a BLAST search (ANIb) were *H. influenzae* KR494 (GenBank accession number CP005967) and >99.9% nucleotide identity. Multilocus sequence typing analysis of the draft genome showed the strain to be sequence type 124, representing *H. influenzae* serotype f (8). After mapping 1,983,017 of the 1,987,874 (99.8%) trimmed reads to the KR494 genome, a total of 666 variants were detected based on a minimum coverage of 7× and a minimum allele frequency of 75%.

Upon examination of these variants, we noticed three discrete regions with a high density of variants, consistent with recombination. These three regions measured 3.6 kb, 3.1 kb, and 7.8 kb in length and contained 135, 133, and 260 of the variants detected (79.3% of all variants). Genes in areas of potential recombination included those encoding IgA-specific serine endopeptidase, *S*-adenosylmethionine (SAM):tRNA ribosyltransferase-isomerase, and a hypothetical protein in the first region; the YjjI family glycine radical enzyme, radical SAM protein, and NupC/NupG family nucleoside CNT transporter in the second region; and the preprotein translocase subunit SecE, ATP-dependent protease ATP-binding subunit ClpX, ATP-dependent Clp protease proteolytic subunit, trigger factor, TonB-dependent receptor, and RelE/StbE family type II toxin-antitoxin system mRNA interferase toxin in the third region. These regions aligned 94 to 97% by nucleotide identity to the *H. influenzae* CGSHiCZ412602 (accession number CP007805) and NCTC8143 (accession number LN831035) strains from the Czech Republic and United Kingdom, respectively.

*H. influenzae* type f has been primarily isolated from cases of pneumonia, sepsis, and meningitis (9). *H. influenzae* is well known to incorporate extracellular DNA; indeed, a laboratory study of *H. influenzae* competence demonstrated 3 to 6 recombination events per transformation of 4 to 12 kb in length, consistent with our data (10). While the spread of *H. influenzae* type f is likely clonal, the recombination ability of this organism provides it a mechanism for continued adaptation (11).

### Accession number(s).

The sequences described in this study are deposited in DDBJ/ENA/GenBank under the accession number NIUV00000000.
